# Improved nutritional value of surplus bread and perennial ryegrass via solid-state fermentation with *Rhizopus oligosporus*

**DOI:** 10.1038/s41538-024-00338-y

**Published:** 2024-11-16

**Authors:** Juan F. Sandoval, Joe Gallagher, Julia Rodriguez-Garcia, Kerry Whiteside, David N. Bryant

**Affiliations:** 1 Institute of Biological, Environmental and Rural Sciences (IBERS), Aberystwyth University, Aberystwyth, UK; 2 Nutrition and Food Science Area, Preventive Medicine and Public Health, Food Science, Toxicology and Forensic Medicine Department, Faculty of Pharmacy, Universitat de València, Burjassot, València Spain; 3 Samworth Brothers Limited, Leicestershire, UK

**Keywords:** Applied microbiology, Proteins

## Abstract

Solid-state fermentation (SSF) is a sustainable method to convert food waste and plant biomass into novel foods for human consumption. Surplus bread crusts (BC) have the structural capacity to serve as an SSF scaffold, and their nutritional value could be increased in combination with perennial ryegrass (PRG), a biorefining feedstock with high-quality protein but an unpleasant sensory profile. SSF with *Rhizopus oligosporus* was investigated with these substrates to determine if the overall nutritional value could be increased. The BC-PRG SSFs were conducted for up to 72 h, over which time the starch content had decreased by up to 89.6%, the total amino acid (AA) content increased by up to 141.9%, and the essential amino acid (EAA) content increased by up to 54.5%. The BC-PRG SSF demonstrated that this process could potentially valorise BC and PRG, both widely available but underexplored substrates, for the production of alternative proteins.

## Introduction

Wheat (*Triticum aestivum*) is one of the most consumed crops worldwide, contributing to 20% of the calories and proteins in human diets^1^, and is the basis of multiple staple foods across different cultures, such as breads, cakes, biscuits, cookies and crackers. Approximately 760 million tons of wheat flour are produced yearly worldwide^2^. Bakery waste, particularly in the form of bread, is a global concern. In 2023, 185 million tons of bread were manufactured^3^, of which roughly 10% was wasted^4^. Some of this waste occurs at the household level, mainly due to spoilage, but also at the manufacture and retail level, where bread waste is called surplus and is primarily comprised of bread crusts and breadcrumbs. While most of this surplus is safe for consumption, it is unsuitable for commercialisation, as it does not comply with the quality specifications given by the manufacturer to the relevant legal authorities, such as the UK Food Standards Agency (FSA), regarding physicochemical composition, and desired appearance and sensory characteristics.

The valorisation of surplus bread has been widely explored. Traditionally, it is either milled to be reincorporated into the production of new batches of bread, or other products such as soups and fried foods^5^, or as an animal feed^6^, but due to the volume of waste generated not all of it is used and ends up being discarded. Ben Rejeb, et al. ^7^ reviewed the available routes for the valorisation of bread waste into value-added products, both through chemical processes and fermentation, including the manufacturing of ethanol, lactic acid, succinic acid, biohydrogen, hydroxymethylfurfural, isolated proteins, pigments, sugar syrups, aromatic compounds, and enzymes. More recently, solid-state fermentation (SSF) has also been explored as an alternative valorisation process. Bread possesses most of the nutrients needed by solid state fermenting microorganisms, specifically filamentous fungi^8^, whilst providing a structural matrix of gluten, the main protein of wheat, and gelatinised starch in which they can grow^9^.

SSF has been used to nutritionally enhance the biomass of cereals, legumes, fruits and vegetables for human consumption^10^, with a low environmental impact. The lack of a liquid phase reduces the generation of wastewater and other potential pollutants^11^, while the use of waste materials as substrates minimises the cost of media preparation, and reduces the energy consumption of the process, as sterilisation is not commonly required^8^. Amongst the multiple filamentous fungi reported in SSF in the food industry, *Rhizopus microsporus* var. *oligosporus (Rmo)* has been used for centuries in the production of tempeh from soybeans (*Glycine max*)^12^. *Rmo* has been shown to valorise surplus bread, reducing the concentration of starch (from 65.8% to 42.7%) while increasing the concentration of crude protein (from 13.2% to 16.1%) and crude fibres (from 11.6% to 26.7%)^13^, and increasing the relative ratio of EAA, minerals and vitamins^14^. *Rmo*, like other filamentous fungi, can produce proteases that cleave protein, thereby altering the AA profile and increasing the concentration of EAA^15^, improving the protein quality. However, as *Rmo* is incapable of nitrogen fixation, increasing the total nitrogen content in the substrate requires external supplementation.

Alternative protein sources with high unexploited potential for human applications are forage grasses and clovers. These crops have been studied as a source of fibres and cellulose^16^, fertilisers^17^, and have been used in biogas, bioethanol^18^, and lactic acid^19^ production, as well as for direct human consumption^20^, however the primary use is as an animal feed. These crops are rich in high-quality protein, in minerals, i.e., magnesium, potassium, phosphorus and calcium^21,22^ and in nutraceutical compounds, i.e., fructan, pinitol, isoflavones and tannins^23^. They can achieve high biomass yields and protein outputs; for instance, a square hectare of land can produce between 1.8 and 3 tons of alfalfa protein, while the same landmass will only produce 150–200 kg of meat protein^24^, with low environmental footprints^25^, which makes them extensively grown worldwide.

Of these crops, PRG (*Lolium perenne)* is one of the most used grass species in the world, especially in Europe where it represents up to 50% of the grass seed market^26^. It’s mainly used as feed in grazing livestock for animal protein production, which compared to using its protein for direct food applications, has been reported to be an inefficient use of the land^27^. It contains a crude protein (CP) content of approximately 12% (dry matter (DM)) and a similarly well-balanced AA profile as soybean^28^, due to the composition of RuBisCO (ribulose-1,5-bisphosphate carboxylase-oxygenase), a key enzyme in the fixation of CO_2_ during photosynthesis, which constitutes up to 50% of the total soluble protein content^29^. Despite this, PRG is not adequate for direct human consumption due to its unpalatability, and its high content of undigestible fibres, particularly cellulose, which can range between 30% and 50% DM^30^. Extraction, fractionation, purification and/or chemical and biological conversion of the protein in forage crops is required for its use in food applications. This has been widely explored by different methods, although with an emphasis on animal feed^29^.

Thus, this research project proposes a novel sustainable process to valorise surplus bread crusts and PRG extracts, a combination of substrates reported here for the first time in the literature. It is hypothesised that the protein content and the concentration of available EAA in the final product will increase by combining these substrates via SSF with *Rmo*. This work provides a fundamental study for the application of forage crop soluble proteins in producing an alternative source of high-quality protein for human consumption via SSF, which would provide useful information for future developments in novel foods.

## Results and discussion

### Solid-State fermentation

Surplus bread crusts (BC) are a relatively dry material (79% DM) with a CP content of 16.3 ± 0.2% DM, crude fibre content of 15.4 ± 0.2% DM and starch content of 69.8 ± 0.3% DM that can be transformed into an alternative food via SSF as part of a strategy in the management of food waste. The porosity of the structure makes the nutrients accessible for *Rmo*, which allows the mycelia to grow throughout the whole substrate and not just superficially, as has been seen in soybeans where the mycelium can penetrate approximately 2 mm into the substrate within 40 h of fermentation^31^. Exploiting the structure and dimensions of BC was fundamental in accelerating the growth of *Rmo*. Firstly, the use of 1 × 1 × 1 cm BC cubes allowed the fungi to grow throughout the substrate (as shown in Fig. S1), in contrast to SSF with milled BC (as shown in Fig. S2) which only supported superficial growth as *Rmo* could not penetrate the substrate. Secondly, the initial substrate moisture of 56% was optimised from a range of 40–70% to the maximum consumption of starch after 72 h of fermentation (as shown in Fig. S3). Thirdly, a pH of 3.5 was selected to avoid the growth of undesirable microorganisms, which was proven to not significantly affect the growth of *Rmo* (as shown in Fig. S4). This adjustment was significant due to the addition of grass juice (GJ), a fresh screw-pressed juice of PRG which contained microorganisms that could influence the process. GJ and dry green solids (GS) were added to increase the nutritional value of the SSF substrate, having CP contents of 16.0 ± 0.7% DM and 34.9 ± 0.3% DM, respectively.

**Fig. 1:**
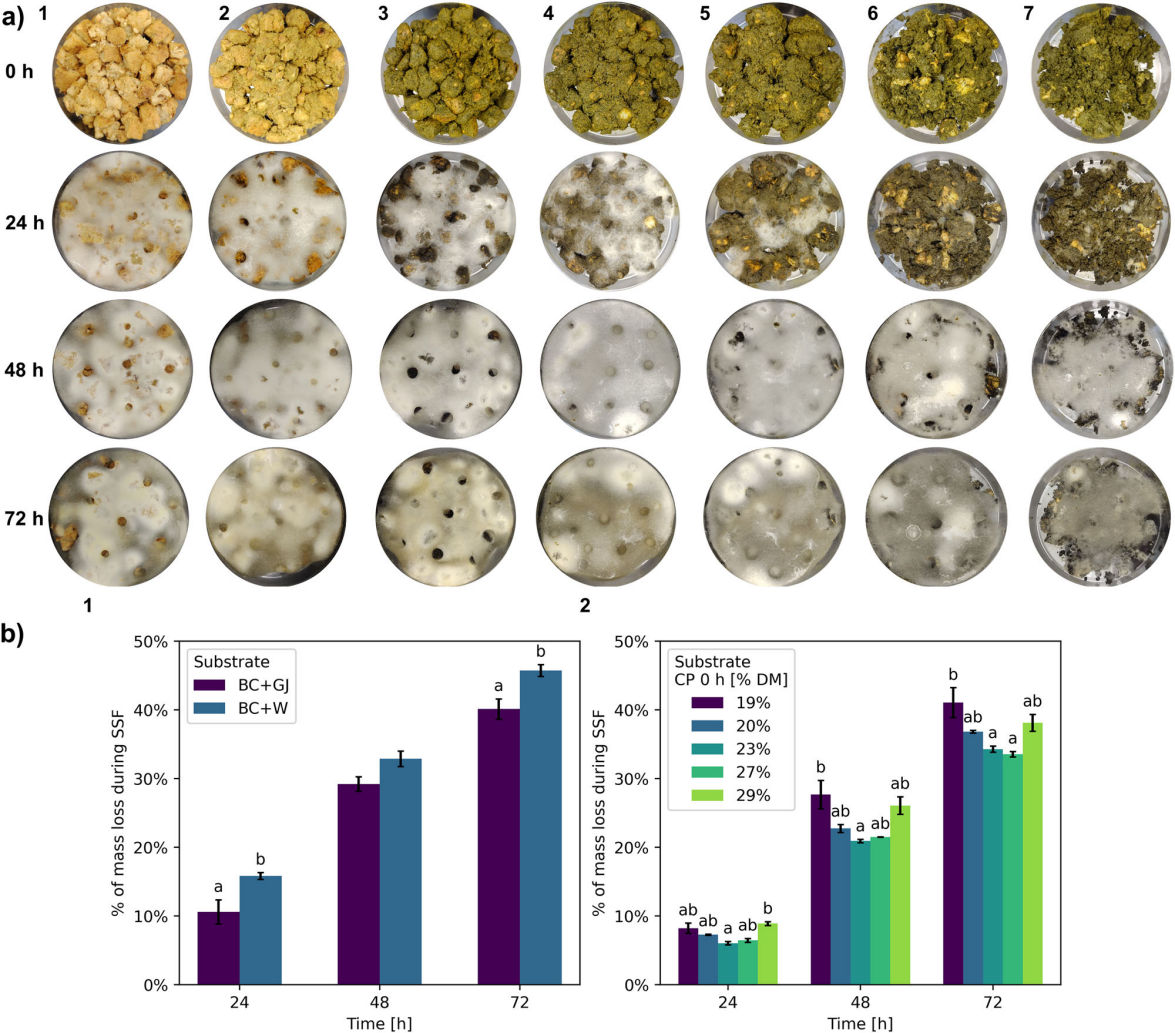
Changes during solid-state fermentation (SSF). **a** Photographic record of SSF experiments. (1) bread crusts (BC)/water (W), (2) BC/green juice (GJ), (3) BC/GJ/dry green solids (GS) to CP 19% DM, (4) BC/GJ/GS to CP 20% DM, (5) BC/GJ/GS to CP 23% DM, (6) BC/GJ/GS to CP 27% DM and (7) BC/GJ/GS to CP 29% DM at 0, 24, 48 and 72 h of fermentation. **b** % of mass loss of substrate at 24, 48 and 72 h of fermentation. (1) BC/GJ and BC/W and (2) BC/GJ/GS to CP 19%, 20%, 23%, 27% or 29% DM. Bars represent the mean value and vertical lines a standard error. Data with different letters in the same time point are significantly different (*p* < 0.05), following Tukey´s HSD post-hoc test. Data without letters are statistically similar.

The first section of experiments, comparing BC/W and BC/GJ, was conducted to provide a baseline of the SSF process and to understand the influence of the plant matrix on *Rmo* (Fig. 1). In contrast, the second section, which compared increasing amounts of GS to increase the CP content, were performed to evaluate alterations in the amino acid profile of the protein after the fermentation.

The SSFs were photographed every 24 h from start to end of the fermentation to record the growth of *Rmo* over the different BC substrates (Fig. 1). Increasing amounts of PRG protein were added to BC (19%, 20%, 23%, 27% or 29% DM) (Fig. 1a, 3–7), and this consistently slowed down the growth of *Rmo* mycelium. After 24 h of SSF and up to 20% DM CP BC/GJ/GS (Fig. 1a, 4), the fungi were able to superficially cover the whole dish. However, higher quantities of GS (BC/GJ/GS to CP 23%, 27% or 29% DM) reduced this growth (Fig. 1a, 5, 6 and 7, respectively). This deceleration could possibly be a consequence of polyphenols in PRG, which have been shown to have antifungal activities in concentrations over 0.2 mg/mL in agar disk diffusion assays^32^. The phenolic content of GJ and GS was measured by LC-MS (Table S1) and found to be of 0.57 $\pm$ 0.01 for GJ and 5.21 $\pm$ 0.06 mg/g DM for GS, which, after their combination with BC prior to SSF, resulted in initial contents of 0.25–2.26 mg/g DM.

After 48 h, *Rmo* mycelium had completely covered all BC/GJ/GS substrates of up to CP 27% DM (Fig. 1 a, 1–6), with BC/GJ/GS to CP 29% DM (Fig. 1a, 7) being partially covered, and started to sporulated, as noted from the grey areas in the white mycelia. After 72 h the fungi continued to sporulate and started to senescence, as seen in the change in colour from white-light grey to yellow-dark grey. This timeframe aligns with the ageing of *Rmo* in SSF and implies the start of the deterioration of the mycelium, the degradation of the nutritional value and negative changes to the sensory profile of the substrate^31^.

**Fig. 2:**
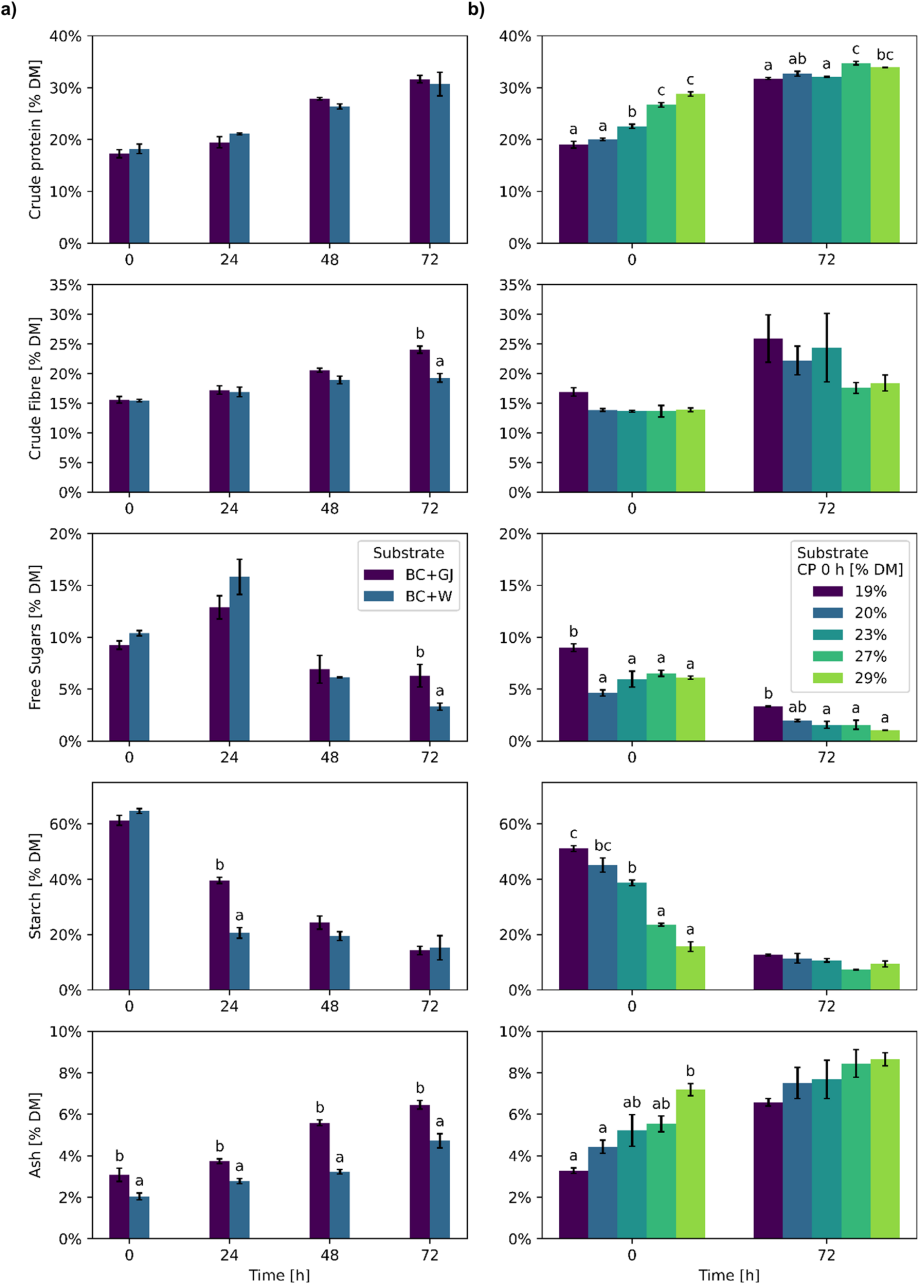
Chemical composition analysis of solid-state fermentation (SSF) samples. **a** Bread crusts (BC)/water (W) and BC/green juice (GJ) at 0, 24, 48 and 72 h of SSF. **b** BC/GJ/dry green solids (GS) to CP 19%, 20%, 23%, 27% and 29% DM at 0 and 72 h of solid-state fermentation (SSF). Bars represent the mean value and vertical lines a standard error. Data with different letters in the same time point are significantly different (*p* < 0.05), following Tukey´s HSD post-hoc test. Data without letters are statistically similar.

Figure 1b details the percentage loss of total substrate mass at 24, 48 and 72 h versus the initial mass at 0 h as an approximate quantitative measure of the growth of the fungi during SSF. A fraction of the mass lost was due to the evaporation of water, while the remainder was attributed to carbohydrate metabolism of the fungi and the production of CO_2_, although the extent by which each contributes to the mass change was not measured. A mathematical model developed by Figueroa-Montero, et al.^33^ in an SFF with *Aspergillus niger* shows the complexity of the mass transfer phenomena in SSF systems, where water and CO_2_ are the main outputs of the fermentation. The mass loss of BC/GJ and BC/W (Fig. 1b, 1) was statistically different across time (*p* = 0.00) and between experimental treatments (*p* = 0.00) at 24 and 72 h, although no interaction was found between the factors (*p* = 0.16) (Table S2), with BC/W having a 49.4% higher loss at 24 h and 14.0% higher loss at 72 h; however, no significant differences in mass loss were observed at 48 h (*p* = 0.08). The mass loss of BC/GJ at different levels of GS (Fig. 1b, 2) had statistical differences across time (*p* = 0.00) and the different experimental treatments (p = 0.00), but no interaction effect was identified (*p* = 0.19) (Table S3), with the highest losses generally occurring at the treatments with 19% and 29% DM and the lowest losses with 23% and 27% DM.

### Chemical composition analysis

**Fig. 3:**
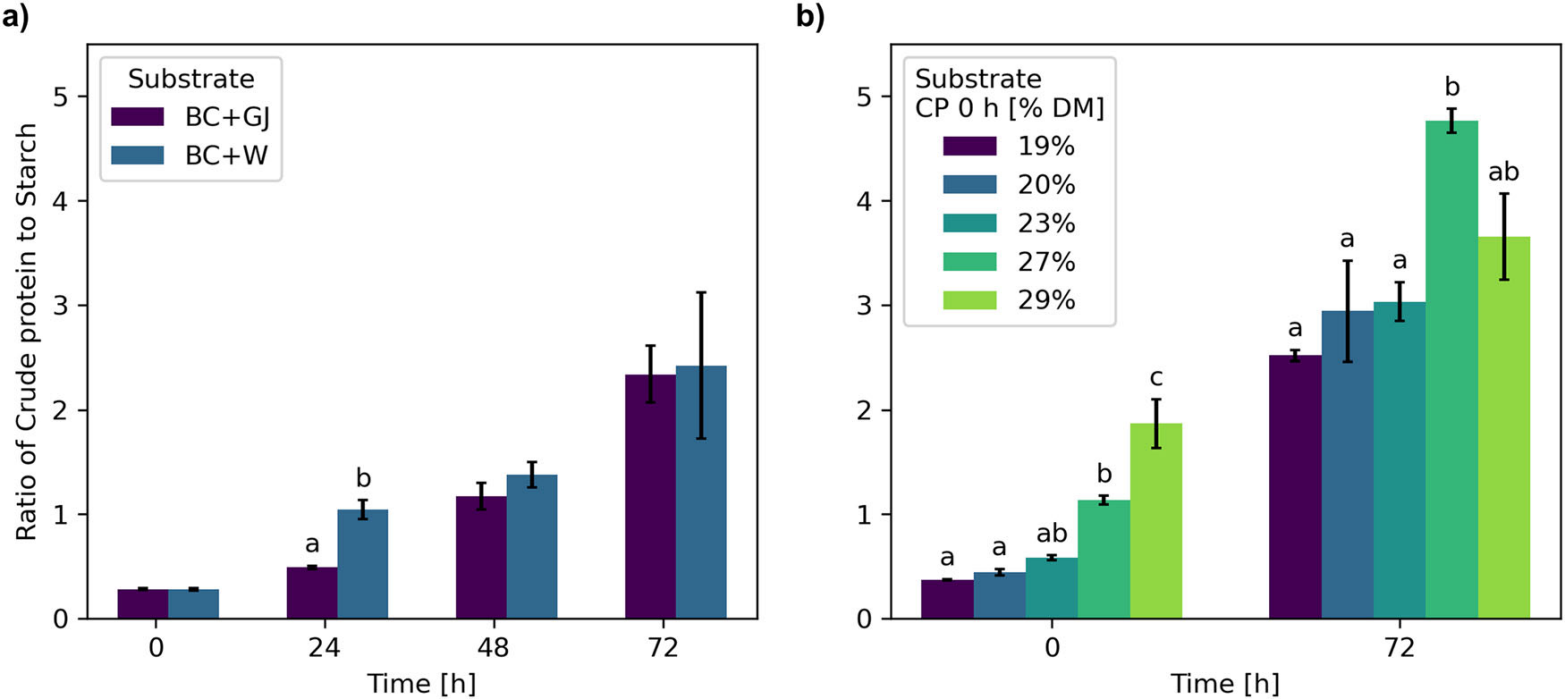
Ratio of crude protein to starch of solid-state fermentation (SSF) samples. **a** Bread crusts (BC)/water (W) and BC/green juice (GJ) at 0, 24, 48 and 72 h of SSF. **b** BC/GJ/dry green solids (GS) to CP 19%, 20%, 23%, 27% and 29% DM at 0 and 72 h of SSF. Bars represent the mean value and vertical lines a standard error. Data with different letters in the same time point are significantly different (*p* < 0.05), following Tukey´s HSD post-hoc test. Data without letters are statistically similar.

The chemical composition of the substrates was assessed to evaluate the change in nutritional properties over the course of the SSF (Fig. 2). The DM content of all experimental units started at 45.7 ± 0.4% and ended at 43.5 ± 1.3%, without substantial differences at any time point. Although the ambient moisture in the SSF chamber was not controlled, the DM was stable throughout the process. The main driver for the mass change in all macronutrients was the digestion of starch as the primary carbon source of the substrate. These changes have also been described in the literature, as fermentation with *Rmo* transforms carbohydrates into fungal biomass, thereby increasing the concentration of crude protein and crude fibre^34^. Additionally, the ratio of crude protein to starch content was calculated to help visualise the changes in the chemical composition in terms of the nutritional value of the substrate (Fig. 3).

Comparing the composition of BC/GJ and BC/W (Fig. 2a), the nutritional value of BC improved considerably in both cases, and few differences were identified. Crude protein content was equal between the experimental treatments (*p* = 0.66) at each time point, but it significantly increased (*p* = 0.00) over time, and no significant interaction was observed between factors (*p* = 0.38) (Table S2). Analysing the change of both treatments over time (Fig. S5) shows an increase in the crude protein content of 74.6% after 72 h of SSF. Crude fibre content was significantly higher (*p* = 0.01) for BC/GJ at 72 h, likely due to the faster carbohydrate metabolism in BC/W that allowed the fungi to use the fibres as the secondary carbon source. After 72 h, crude fibre increased by 54.2% in BC/GJ and by 24.8% in BC/W. Starch content was significantly lower (*p* = 0.00) for BC/W at 24 h (Fig. 1b, 1) due to a faster metabolic activity during the first hours of the fermentation, as seen by the higher free sugar content at the same time point as a result of the enzymatic hydrolysis of starch into smaller oligosaccharides and monosaccharides. After 48 and 72 h, the free sugar content is reduced as monosaccharides are used as the main carbon source for fungal growth and transformed into water and CO_2_. After 72 h, the starch content is reduced by 76.8% in BC/GJ and by 76.5% in BC/W. Ash content was significantly higher (*p* = 0.00) for BC/GJ in all-time points likely due to the added minerals, such as phosphorus, potassium, calcium and magnesium, present in GJ, as reported previously in the literature^35^. At the end of the SSF, the ratio of CP to starch increased from 0.3 to 2.3 (Fig. 3a) which, similar to the changes in crude protein, was significantly different (*p* = 0.00) across time but not significantly different (*p* = 0.39) between the experimental treatments (Table S2). As a whole, these results indicated the feasibility of adding forage crop extracts to the SSF process and led to the increasing levels of forage crop protein of the next set of experiments. These changes induced modifications to the flavour profile of the substrates, particularly in BC/GJ, as the material developed sweet notes reminiscent of pineapple and lost all notes related to the green smell of grass, as it was corroborated in informal sensory tests. After 72 h, the senescence of *Rmo* (Fig. 1 a) developed undesirable pungent smells akin to the growth of the black bread mould *R. stolonifer*, the most common household bread mould. SPME GC-MS olfactory analysis was carried out over SSF samples to corroborate these findings, but a complete analysis was outside the scope of this publication. Another manuscript detailing these findings is under preparation.

The composition of the BC/GJ/GS samples (Fig. 2 b) was influenced by the amount of GS added, as higher GS content resulted in a lower concentration of starch and a higher concentration of CP in the substrate while maintaining the same initial moisture. This led to statistical differences for all components (*p* < 0.05) between experimental treatments and across time, except for crude fibre between the experimental treatments (*p* = 0.20), and significant interactions (*p* < 0.05) between the factors for crude protein, free sugars and starch (Table S3). As the starch content decreased to 10.3 ± 0.8% DM after 72 h from initial contents of 51.1 ± 1.1% DM to 15.6 ± 1.7% DM at 0 h, the rest of the components were concentrated. After 72 h, only crude protein and free sugar content were statistically different (*p* < 0.05) among treatments, while crude fibre, starch and ash content were similar (*p* > 0.05). At this point, crude protein content increased by 67.3% in BC/GJ/GS CP 19% DM. Higher GS levels resulted in lower CP content increases after SSF, achieving a 17.8% increase at BC/GJ/GS CP 29% DM. The ratio of CP to starch content was significantly higher (*p* = 0.00) in BC/GJ/GS CP 27% and 29% DM after 72 h (Fig. 3b) than in the other treatments with GS. As with the BC/W and BC/GJ experiments, the change in the free sugar content also induced changes to the substrate aroma, reducing the characteristic grassy smell, as noticed after informal testing.

At 72 h the CP composition of all the fermented substrates was statistically higher (*p* = 0.00) than those of unfermented BC (Fig. 2a, time 0, BC + W) and had a maximum increase in CP of 113.1% in BC/GJ/GS CP 27% DM mirrored by a significant decrease in starch content (*p* = 0.00) of 89.6% (Table S4).

### Amino acid profile analysis

Essential (EAA) and non-essential (NEAA) amino acids were analysed by HPLC to evaluate the transformation of the amino acid profile of the BC substrates over time. All EAA, except for tryptophan, were quantified and their concentrations are shown as a %DM, as well as the sum of the total amino acids (AA), sum of EAA, sum of NEAA, the percentage of EAA over total AA and NEAA over total AA (Tables 1 and 2). The biotransformation of the protein of the substrate, as an effect of the metabolism of *Rmo* during the SSF, led to improved amino acid profiles.

**Table 1:** Total amino acid profile [%DM] from bread crusts (BC)/water (W) and BC/green juice (GJ) at 0, 24, 48 and 72 h of solid-state fermentation (SSF)

Substrate	BC/W				BC/GJ			
Composition	0 h	24 h	48 h	72 h	0 h	24 h	48 h	72 h
EAA								
Histidine	0.38 ± 0.01^b^	0.37 ± 0.02^a^	0.49 ± 0.01^a^	0.58 ± 0.02^b^	0.30 ± 0.02^a^	0.31 ± 0.01^a^	0.52 ± 0.03^a^	0.57 ± 0.07^a^
Isoleucine	0.45 ± 0.01^b^	0.57 ± 0.05^a^	0.79 ± 0.02^a^	0.85 ± 0.06^a^	0.42 ± 0.07^a^	0.63 ± 0.02^a^	0.79 ± 0.07^a^	0.78 ± 0.13^a^
Leucine	0.91 ± 0.02^a^	0.97 ± 0.08^a^	1.25 ± 0.03^a^	1.35 ± 0.07^a^	0.85 ± 0.04^a^	0.98 ± 0.02^a^	1.16 ± 0.11^a^	1.26 ± 0.11^a^
Lysine	0.54 ± 0.25^a^	0.99 ± 0.38^a^	1.20 ± 0.40^a^	1.41 ± 0.21^a^	0.22 ± 0.01^a^	0.34 ± 0.02^a^	0.95 ± 0.07^a^	0.93 ± 0.19^a^
Methionine	0.24 ± 0.02^b^	0.29 ± 0.07^a^	0.33 ± 0.04^b^	0.25 ± 0.04^a^	0.19 ± 0.05^a^	0.13 ± 0.02^a^	0.20 ± 0.03^a^	0.31 ± 0.06^a^
Phenylalanine	0.58 ± 0.01^a^	0.62 ± 0.04^a^	0.74 ± 0.01^a^	0.74 ± 0.03^a^	0.66 ± 0.02^b^	0.71 ± 0.02^a^	0.82 ± 0.07^a^	0.96 ± 0.06^b^
Threonine	0.40 ± 0.01^a^	0.51 ± 0.03^a^	0.71 ± 0.01^a^	0.80 ± 0.05^a^	0.37 ± 0.01^a^	0.50 ± 0.02^a^	0.77 ± 0.06^a^	0.87 ± 0.07^b^
Valine	0.78 ± 0.04^b^	1.02 ± 0.16^a^	1.41 ± 0.08^b^	1.53 ± 0.13^a^	0.56 ± 0.03^a^	0.72 ± 0.02^a^	0.93 ± 0.08^a^	1.02 ± 0.09^a^
NEAA								
Alanine	0.49 ± 0.01^b^	0.80 ± 0.08^a^	1.30 ± 0.06^a^	1.78 ± 0.21^a^	0.43 ± 0.01^a^	0.75 ± 0.02^a^	1.15 ± 0.09^a^	1.29 ± 0.10^a^
Aspartic acid	0.57 ± 0.01^a^	1.06 ± 0.07^a^	1.80 ± 0.04^a^	1.87 ± 0.17^a^	0.67 ± 0.01^b^	1.01 ± 0.05^a^	1.65 ± 0.11^a^	1.99 ± 0.09^a^
Cysteine	0.05 ± 0.01^a^	0.04 ± 0.00^a^	0.07 ± 0.01^a^	0.09 ± 0.01^a^	0.06 ± 0.02^b^	0.03 ± 0.00^a^	0.04 ± 0.00^a^	0.10 ± 0.02^a^
Glutamic acid	4.63 ± 0.07^a^	3.94 ± 0.28^a^	4.53 ± 0.12^b^	4.11 ± 0.16^a^	5.11 ± 0.31^a^	3.87 ± 0.07^a^	3.42 ± 0.30^a^	4.14 ± 0.13^a^
Glycine	0.36 ± 0.01^a^	0.43 ± 0.03^a^	0.62 ± 0.05^a^	0.67 ± 0.05^a^	0.53 ± 0.03^b^	0.73 ± 0.02^b^	1.07 ± 0.07^b^	1.10 ± 0.17^b^
Proline	1.58 ± 0.04^a^	1.48 ± 0.13^a^	1.78 ± 0.03^b^	1.65 ± 0.08^a^	1.47 ± 0.02^a^	1.34 ± 0.02^a^	1.19 ± 0.12^a^	1.42 ± 0.05^a^
Serine	0.58 ± 0.01^a^	0.59 ± 0.04^a^	0.74 ± 0.02^a^	0.81 ± 0.04^a^	0.61 ± 0.03^a^	0.62 ± 0.00^a^	0.80 ± 0.07^a^	0.99 ± 0.04^b^
Tyrosine	0.68 ± 0.09^b^	0.90 ± 0.20^a^	1.05 ± 0.17^b^	1.11 ± 0.10^b^	0.32 ± 0.11^a^	0.26 ± 0.00^a^	0.46 ± 0.03^a^	0.70 ± 0.07^a^
Arginine	0.67 ± 0.01^b^	0.71 ± 0.04^a^	1.01 ± 0.06^a^	1.15 ± 0.06^a^	0.47 ± 0.03^a^	0.56 ± 0.02^a^	0.95 ± 0.06^a^	1.08 ± 0.10^a^
Sum AA	13.88 ± 0.48^a^	15.31 ± 1.65^a^	19.84 ± 1.03^a^	20.75 ± 1.38^a^	13.23 ± 0.37^a^	13.50 ± 0.18^a^	16.85 ± 1.28^a^	19.51 ± 1.10^a^
Sum EAA	4.26 ± 0.32^a^	5.36 ± 0.81^a^	6.93 ± 0.59^a^	7.51 ± 0.58^a^	3.57 ± 0.15^a^	4.32 ± 0.15^a^	6.13 ± 0.48^a^	6.70 ± 0.65^a^
Sum NEAA	9.61 ± 0.18^a^	9.95 ± 0.86^a^	12.91 ± 0.46^a^	13.24 ± 0.81^a^	9.67 ± 0.42^a^	9.17 ± 0.03^a^	10.72 ± 0.80^a^	12.81 ± 0.46^a^
% EAA/AA	30.63 ± 1.50^a^	34.63 ± 0.68^a^	34.80 ± 0.36^a^	36.15 ± 1.49^a^	27.07 ± 1.27^a^	32.03 ± 1.94^a^	36.38 ± 1.16^a^	34.00 ± 0.47^a^
% NEAA/AA	69.37 ± 1.50^a^	65.37 ± 0.68^a^	65.20 ± 0.36^a^	63.85 ± 1.49^a^	72.93 ± 1.27^a^	67.97 ± 1.94^a^	63.62 ± 1.16^a^	66.00 ± 0.47^a^

Results are expressed as the mean value ± a standard error. Data with different letters in the same time point are significantly different (*p* < 0.05) between BC/W and BC/GJ, following Tukey´s HSD post-hoc test. Data with the same letters are statistically similar.

*CP* crude protein, *AA* amino acids, *EAA* essential amino acids, *NEAA* non-essential amino acids.

**Table 2:** Total amino acid profile [%DM] from bread crusts (BC)/green juice (GJ)/dry green solids (GS) to CP 19%, 20%, 23%, 27% and 29% DM at 0 and 72 h of solid-state fermentation (SSF)

CP 0 h [%DM]	19%		20%		23%		27%		29%	
Composition	0 h	72 h	0 h	72 h	0 h	72 h	0 h	72 h	0 h	72 h
EAA										
Histidine	0.22 ± 0.04^a^	0.43 ± 0.05^a^	0.37 ± 0.05^a^	0.81 ± 0.00^b^	0.54 ± 0.03^a^	1.66 ± 0.03^c^	0.90 ± 0.32^a^	0.95 ± 0.00^b^	1.88 ± 0.03^b^	1.41 ± 0.15^c^
Isoleucine	0.30 ± 0.05^a^	0.59 ± 0.01^a^	0.51 ± 0.01^b^	0.71 ± 0.02^ab^	0.66 ± 0.03^bc^	0.79 ± 0.04^ab^	0.75 ± 0.04^c^	0.90 ± 0.01^b^	1.06 ± 0.05^d^	1.23 ± 0.10^c^
Leucine	0.79 ± 0.14^a^	1.22 ± 0.00^a^	1.21 ± 0.05^ab^	1.47 ± 0.01^a^	1.46 ± 0.06^b^	1.58 ± 0.07^ab^	1.59 ± 0.05^b^	1.87 ± 0.03^b^	2.23 ± 0.12^c^	2.38 ± 0.15^c^
Lysine	0.30 ± 0.06^a^	0.60 ± 0.01^a^	0.45 ± 0.01^a^	0.77 ± 0.09^a^	0.62 ± 0.03^b^	0.46 ± 0.02^a^	0.64 ± 0.03^b^	0.75 ± 0.00^a^	0.64 ± 0.02^b^	1.34 ± 0.20^b^
Methionine	0.33 ± 0.01^a^	0.48 ± 0.01^a^	0.42 ± 0.01^a^	0.49 ± 0.02^a^	0.51 ± 0.03^ab^	0.65 ± 0.00^a^	0.62 ± 0.06^b^	0.69 ± 0.08^b^	0.66 ± 0.04^b^	0.76 ± 0.01^b^
Phenylalanine	0.61 ± 0.09^a^	0.96 ± 0.07^a^	0.98 ± 0.07^ab^	1.08 ± 0.05^a^	1.21 ± 0.07^ab^	2.45 ± 0.08^b^	1.60 ± 0.23^b^	2.28 ± 0.18^b^	2.85 ± 0.12^c^	2.26 ± 0.23^b^
Threonine	0.37 ± 0.07^a^	0.82 ± 0.02^a^	0.61 ± 0.04^ab^	0.90 ± 0.01^a^	0.79 ± 0.05^bc^	1.38 ± 0.06^b^	0.94 ± 0.08^c^	1.31 ± 0.01^b^	1.53 ± 0.05^d^	1.53 ± 0.08^b^
Valine	0.72 ± 0.10^a^	0.89 ± 0.01^a^	0.78 ± 0.01^ab^	0.97 ± 0.02^a^	1.01 ± 0.04^ab^	0.99 ± 0.00^a^	1.21 ± 0.13^b^	1.31 ± 0.23^a^	1.31 ± 0.03^b^	1.62 ± 0.01^b^
NEAA										
Alanine	0.48 ± 0.11^a^	1.25 ± 0.04^a^	0.77 ± 0.03^ab^	1.45 ± 0.01^a^	0.97 ± 0.03^bc^	1.21 ± 0.09^a^	1.11 ± 0.06^c^	1.34 ± 0.04^a^	1.46 ± 0.02^d^	1.98 ± 0.23^b^
Aspartic acid	0.86 ± 0.21^a^	2.15 ± 0.26^a^	1.32 ± 0.02^ab^	2.33 ± 0.03^a^	1.71 ± 0.08^b^	1.75 ± 0.13^a^	1.77 ± 0.18^b^	1.74 ± 0.13^a^	2.00 ± 0.07^b^	3.41 ± 0.29^b^
Cysteine	0.11 ± 0.03^a^	0.14 ± 0.01^a^	0.13 ± 0.00^a^	0.14 ± 0.01^a^	0.10 ± 0.00^a^	0.20 ± 0.01^a^	0.14 ± 0.04^a^	0.15 ± 0.03^a^	0.17 ± 0.01^a^	0.19 ± 0.02^a^
Glutamic acid	4.41 ± 0.71^a^	3.65 ± 0.37^ab^	5.01 ± 0.31^a^	3.63 ± 0.02^ab^	4.61 ± 0.03^a^	2.87 ± 0.17^a^	3.48 ± 0.48^a^	2.56 ± 0.20^a^	3.40 ± 0.16^a^	4.47 ± 0.33^b^
Glycine	0.42 ± 0.08^a^	0.77 ± 0.00^a^	0.67 ± 0.02^ab^	0.94 ± 0.10^a^	0.81 ± 0.02^b^	2.68 ± 1.42^a^	0.92 ± 0.06^b^	1.31 ± 0.01^a^	1.47 ± 0.06^c^	1.45 ± 0.12^a^
Proline	1.20 ± 0.19^a^	1.20 ± 0.02^a^	1.50 ± 0.00^a^	1.27 ± 0.06^a^	1.57 ± 0.00^a^	1.50 ± 0.02^a^	1.49 ± 0.05^a^	1.41 ± 0.01^a^	1.64 ± 0.04^a^	2.12 ± 0.44^a^
Serine	0.55 ± 0.10^a^	0.96 ± 0.01^a^	0.81 ± 0.04^ab^	1.00 ± 0.01^a^	0.96 ± 0.04^b^	1.46 ± 0.06^b^	1.03 ± 0.04^b^	1.34 ± 0.00^b^	1.57 ± 0.03^c^	1.53 ± 0.08^b^
Tyrosine	0.64 ± 0.01^a^	0.93 ± 0.03^a^	0.80 ± 0.02^a^	0.95 ± 0.02^a^	1.02 ± 0.11^ab^	1.45 ± 0.03^a^	1.38 ± 0.20^b^	1.82 ± 0.45^a^	1.49 ± 0.09^b^	1.78 ± 0.02^a^
Arginine	0.44 ± 0.06^a^	1.05 ± 0.13^a^	0.84 ± 0.11^a^	1.34 ± 0.04^a^	1.18 ± 0.12^ab^	2.68 ± 0.09^b^	1.65 ± 0.29^b^	2.33 ± 0.01^b^	3.16 ± 0.05^c^	2.58 ± 0.09^b^
Sum AA	12.77 ± 1.79^a^	18.11 ± 0.31^a^	17.18 ± 0.12^ab^	20.25 ± 0.27^ab^	19.74 ± 0.72^b^	25.76 ± 0.76^b^	21.23 ± 0.97^b^	24.06 ± 0.54^b^	28.51 ± 0.18^c^	32.01 ± 2.21^c^
Sum EAA	3.64 ± 0.33^a^	6.02 ± 0.16^a^	5.32 ± 0.22^ab^	7.20 ± 0.04^a^	6.81 ± 0.35^bc^	9.95 ± 0.26^b^	8.26 ± 0.89^c^	10.07 ± 0.46^b^	12.17 ± 0.07^d^	12.52 ± 0.64^c^
Sum NEAA	9.13 ± 1.46^a^	12.09 ± 0.47^a^	11.86 ± 0.10^ab^	13.05 ± 0.23^a^	12.94 ± 0.38^bc^	15.80 ± 1.01^ab^	12.97 ± 0.09^bc^	13.99 ± 0.07^a^	16.34 ± 0.12^c^	19.50 ± 1.58^b^
% EAA/AA	28.72 ± 1.43^a^	33.24 ± 1.46^a^	30.94 ± 1.07^a^	35.56 ± 0.27^ab^	34.45 ± 0.49^ab^	38.71 ± 2.13^ab^	38.80 ± 2.40^bc^	41.83 ± 0.99^b^	42.69 ± 0.04^c^	39.15 ± 0.72^ab^
% NEAA/AA	71.28 ± 1.43^a^	66.76 ± 1.46^b^	69.06 ± 1.07^a^	64.44 ± 0.27^ab^	65.55 ± 0.49^ab^	61.29 ± 2.13^ab^	61.20 ± 2.40^bc^	58.17 ± 0.99^a^	57.31 ± 0.04^c^	60.85 ± 0.72^ab^

Results are expressed as the mean value ± a standard error. Data with different letters in the same time point are significantly different (*p* < 0.05) between the different levels of GS, following Tukey´s HSD post-hoc test. Data with the same letters are statistically similar.

*CP* crude protein, *DM* dry matter, *AA* amino acids, *EAA* essential amino acids, *NEAA* non-essential amino acids.

In all experimental units, although to a lesser extent in the GS experiments due to the lower per cent composition of BC in the substrates, glutamic acid was the AA with the highest concentration at 0 h (from 3.40 to 5.11%DM), as this is the predominant AA in wheat protein. Whilst this AA contributes to a pleasing savoury flavour, it is not nutritionally desirable as it is a NEAA. Coupled with the low concentration of lysine versus the FAO nutritional guidelines^36^, wheat protein as a whole has a low protein digestibility-corrected amino acid score (PDCAAS) of 0.42^37^ and thus not considered a high-quality protein for human nutrition. The positive effect of fungal metabolism over the AA profile can be seen in the BC/W SSF (Table 1) with the AA concentrations that were significantly affected by time (Table S2). The percentage of EAA over the total AA increased from 30.63 ± 1.5% to 36.15 ± 1.5%, a significant 18.0% increase (*p* = 0.00) after 72 h. At this latter time point, the AA with the greatest significant increases (*p* = 0.00) over time were threonine (101.7%), alanine (263.0%%) and aspartic acid (225.1%) while the AA with the greatest significant decrease (*p* = 0.00) was glutamic acid (−11.4%) (Table S2). The bioconversion of certain NEAA, such as glutamic acid, has been studied via metabolic pathways such as the $\alpha$-aminoadipate pathway, where glutamic acid is the primary amine source for the production of lysine, which selectively allows fungal metabolism to increase the composition of EAA by depleting NEAA^38^. The variation in total AA was significant (*p* = 0.00) over time, (Table S2) as expected from the increase in CP, but the ratio of total AA to CP decreases over time, from 76.49 ± 2.2 at 0 h to 67.65 ± 0.7 g AA/100 g CP at 72 h, which can be explained both by the continuous activity of fungal proteases, which reduces functional protein to nitrogen subproducts, and the incorporation of nitrogen into the cell walls of *Rmo* in the form of chitin during its growth. Comparing BC/GJ with BC/W (Table 1) yields differences in the AA profile. The relatively slower fermentation of BC/GJ (Fig. 1) produces a maximum percentage of EAA at 48 h (36.4%), instead of 72 h (34.0%) as in BC/W, but the total sum of AA is otherwise similar (*p* = 0.09). Comparing the contents of AA after 72 h of BC/GJ with those of BC/W, BC/GJ had a higher content of methionine, phenylalanine, threonine, cysteine, glycine and serine (Table 1).

Significant differences that show the effect of forage protein over the AA profile can be seen in the experiments with BC/GJ/GS (Table 2). Increasing additions of GS not only reduces the glutamic acid content at 0 h but also reduces proline and increases all EAA. Of particular relevance to the nutritional quality of BC, the increase of isoleucine, leucine, lysine and methionine, all present in *L. perenne*^28^, greatly covers the AA deficiencies of wheat. Also, at 0 h, the sum of total AA increased from 12.77 ± 1.8 at the lowest level of GS up to 28.51 ± 0.2%DM at the highest, a 123.3% increase, and the percentage of EAA increased from 28.72 ± 1.4 up to 42.69 ± 0.0%, a 48.6% increase. After 72 h of SSF, the sum of total AA significantly increased (*p* = 0.00) in all experiments between 12.3 and 41.8%, while the percentage of EAA increased by 48.6% at the lowest level of GS and decreased by 8.3% at the highest level of GS, primarily due to the increase in NEAA such as aspartic acid and glutamic acid. While all EAA were affected throughout the SSF by the addition of GS (Table 2), only histidine, lysine, phenylalanine and threonine were affected by the interaction of both the addition of GS and time (*p* < 0.05), showing the specific metabolic activity of *Rmo* towards certain AA of interest, such as lysine (Table S3). At 72 h the AA profile of the substrates with added GS was significantly different (*p* = 0.00) than those of unfermented BC (Table S4). The SSF with BC/GJ/GS CP 29% DM had the highest sum of total AA, a 141.9% increase in comparison to unfermented BC, while the SSF with BC/GJ/GS CP 27% DM had the highest percentage composition of EAA, a 54.5% increase in comparison to unfermented BC.

The change of each AA after the SSF varied considerably between each level of GS, likely in response to complex shifts in the metabolic pathways taken by *Rmo* during SSF, but local maxima and minima can be identified. For instance, the greatest increase in histidine occurs in BC/GJ/GS CP 23% DM (206.4%), of lysine in CP 29% DM (109.5%) and of threonine in CP 19% DM (157.5%), which opens the possibility of targeting the concentration of particular AA at different substrate compositions. Alongside the changes in the proximal composition (Fig. 2b), this shows that supplementing the CP content of BC with GS results in similar levels of CP (32.5 ± 0.5%) after 72 h of SSF while increasing both the composition of total AA and the percentage composition of EAA.

The impact of these changes for food applications can be further seen in Table 3, which shows the EAA reference ratios calculated against the FAO nutritional guidelines for adults^36^. Currently, calculation of the nutritional quality of protein is performed via the PDCAAS method, which multiplies the lowest reference ratio from the EAA list with the in-vitro digestibility of the protein. This method undervalues proteins that are deficient in particular AAs, such as lysine in wheat, and ignores AAs far in excess of the reference. The reference ratios of the EAA in the SSF substrates after 72 h of fermentation increase compared to those of unfermented BC, with the exception of isoleucine, leucine and lysine in BC/GJ/GS 19–27% DM CP and of methionine + cysteine in BC/W. As neither tryptophan nor the changes in the digestibility of the protein were quantified a PDCAAS could not be calculated, but the ratios suggest that BC/GJ/GS 29% DM CP would have the highest value, as the lowest reference ratio is still for lysine, but is of 0.88 compared to the 0.65 of unfermented BC. In this same SSF sample, all other reference ratios are over 1.0, with particularly high increments in ratio for histidine (2.78) and phenylalanine + tyrosine (3.97). Reports of the changes in the protein digestibility after SSF with *Rhizopus* sp. are mixed in the literature^39,40^, and as discussed in this manuscript, could be affected by the phenolic content of PRG and the changes in the free amino acid content of the substrate after SSF.

**Table 3:** Essential amino acid (EAA) reference ratios calculated against the FAO nutritional guidelines [g/100 g CP] for adults for unfermented bread crusts (BC), and solid-state fermentation (SSF) substrates BC/water (W), BC/green juice (GJ), BC/GJ/green solids (GS) to CP 19%, 20%, 23%, 27% and 29% DM at 72 h

EAA	FAO requirement	BC	Substrate after 72 h SSF						
			BC/W	BC/GJ	BC/GJ/GS to % DM CP				
					19%	20%	23%	27%	29%
Histidine	1.5	1.38	1.27	1.32	0.91	1.65	3.45	1.82	2.78
Isoleucine	3	0.82	0.93	0.89	0.62	0.73	0.82	0.87	1.21
Leucine	5.9	0.85	0.75	0.76	0.65	0.76	0.83	0.91	1.19
Lysine	4.5	0.65	1.01	0.70	0.42	0.52	0.32	0.48	0.88
Threonine	2.3	0.95	1.13	1.33	1.13	1.19	1.87	1.64	1.96
Valine	3	1.43	1.66	1.20	0.94	0.99	1.03	1.26	1.59
Methionine + Cysteine	2.2	0.72	0.50	0.71	0.89	0.87	1.21	1.11	1.27
Phenylalanine + Tyrosine	3	2.30	2.02	2.00	1.99	2.08	4.04	3.93	3.97
Tryptophan	0.6								

*CP* crude protein, *DM* dry matter.

This research establishes the viability and nutritional potential of combining forage crops and bakery surplus to reduce waste, improve circularity within the food industry and upgrade non-conventional crops for further development as human food. As PRG is not currently considered a common food source and is thus classified as a novel food, several aspects of food safety need to be explored further before its potential use as a protein source, such as the presence of microbial, and chemical hazards. The microbial hazards mostly include lactic acid bacteria but depending on the quality of the crop and the time between harvest and biorefinery, several other species of pathogenic bacteria such as *Clostridium sp*. and *Bacillus sp*. may appear. Chemical hazards include heavy metals accumulated in the crops during their growth and fertilizers present in the soil^41^. The microbial burden, although controlled in SSF by reducing the pH, could be reduced with by using the same methods applied to sanitise salad vegetables, such as chlorine solutions, calcined calcium, and hydrogen peroxide, among others^42^. Overall, this study provides novel insights for the potential of other combinations of substrates to be fermented with *Rmo*, or other food-safe filamentous fungi used in SSF, such as *Aspergillus* sp. and *Neurospora* sp. and explores the changes in the nutritional quality and sensory profile. Industrially, this provides the agricultural sector an additional route to valorise their crops through a biorefinery, and information for the food manufacturing sector to develop novel alternatives for animal proteins with lower environmental impacts, which would reduce food waste, increase food security, and decarbonize the food system. The resulting fermented products could thus be explored as novel foods, or the processed protein-rich powder incorporated in the preparation of wheat-based staple foods such as bread, cookies, pasta, pastries and cakes, by replacing fractions of wheat flour, which requires further research.

## Materials and methods

### Raw materials

Acetonitrile, formic acid, ammonium formate, methanol, ethanol and sodium hydroxide were purchased from Fischer Scientific UK Ltd (Leicestershire, UK). Phosphoric acid, phenol, DL-norvaline, potato dextrose agar and tween 80 were purchased from Merck Life Science UK Ltd (Dorset, UK). Anhydrous acetonitrile, hydrochloric acid, borate buffer and amino acid standard H were purchased from ThermoFischer Scientific Ltd (Paisley, UK). 6-Aminoquinolyl-N-hydroxysuccinimidyl carbamate (AQC) was purchased from Synchem UG & Co. KG (Altenburg, Germany). Total starch analysis kit was purchased from Megazyme Ltd (Wicklow, Ireland). Unless specified, all this study’s chemicals, solvents and reagents were of at least analytical grade. For HPLC, the AccQ-Tag Amino acids C18 column was purchased from Waters Ltd (Wilmslow, UK), and the REZEX-ROA Organic acid H+ (8%) Ion exclusion column was purchased from Phenomenex Inc (CA, US) For LC-MS, the Nova-Pak C18 Column was purchased from Waters Ltd (Wilmslow, UK). Bread crusts (BC) from surplus wheat flour loaf bread were provided by Bradgate Bakery (a division of Samworth Brothers Ltd, Leicestershire, UK) and manually cut to approximately 1 × 1 × 1 cm cubes.

### Perennial ryegrass harvesting and pilot-scale processing

Perennial ryegrass (*Lolium perenne*) was seeded in 2021 in a farmed experimental plot at Aberystwyth University in Aberystwyth, UK and harvested in June 2022. 1 ton of freshly harvested PRG was screw pressed at pilot scale in a CP-10 screw press (Vincent corporation, FL, US) to obtain 410 L of GJ with a pH of 5.62 (adjusted to pH 3.5 with H_3_PO_4_) and frozen at −20 °C in 2 L aliquots to avoid the growth of undesired microorganisms. The resulting GJ had a DM content of 4.98%, and CP content of 16.0% DM. 32 L of GJ were clarified using a GLE continuous centrifuge (Carl Padberg Zemtrofugenbau GmbH, Lahr, Germany) at 38,000 × *g* to obtain 800 g of wet protein-rich precipitate with a DM content of 27.3%, which was freeze-dried to a GS with final DM content of 85.0% and CP content of 34.9% DM.

### Microorganism and inoculum preparation

*Rmo* spores were purchased from fermentationculture.eu and preserved in 10% glycerol solution at −80 °C and in potato dextrose agar (PDA) plates at 32 °C. After 5 days of growth at 32 °C *Rmo* spores were harvested from PDA plates in 50 mL sterile distilled water (0.03% tween 80) and counted in a Neubauer chamber to approximately $1\times {10}^{7}$ spores/mL and SSFs were inoculated at $2\times {10}^{5}$ spores/g substrate.

### Solid-state fermentation

SSF experiments were conducted in sterile 100 mm diameter Petri dishes. The experiments were divided into two sequential sections: firstly, 25 g of BC were adjusted to a moisture content of 56% (v/w DM) with either sterile distilled water (W) or GJ and fermented for 24, 48 or 72 h. These experiments were done in triplicate for a total of *n* = 24 samples. Secondly, 25 g of BC were adjusted to a moisture content of 56% (v/w DM) with GJ, supplemented with GS to final substrate CP contents of 19%, 20%, 23%, 27% or 29% DM, and fermented for 72 h. These experiments were done in duplicate for a total of *n* = 24 samples. For each fermentation, 40 g of mixed substrate (BC with W/GJ/GS), at pH 3.5 (adjusted with H_3_PO_4_), covered with lids but not closed hermetically to enable airflow, were incubated at 32 °C, having been weighed and photographed before and after the SSF. All fermented samples were dried in a vacuum oven at 45 °C, milled to a fine powder and then stored at 4 °C for further analysis.

### Chemical composition analysis

The moisture content of the samples was determined according to AOAC method 930.15^43^. Ash content was determined according to AOAC method 942.05^43^. Crude protein content was determined using the Dumas combustion method in a Vario MAX Cube CN analyser (Elementar, Stockport, UK). A conversion factor of 6.25 was used to convert total nitrogen to crude protein content. Crude fibre content was determined according to the AOCS method Ba 6a-05^44^ in an ANKOM A2000 fibre analyser (ANKOM Technology, NY, US). Free sugars (glucose, fructose and sucrose) were determined by mixing 200 mg of sample with 1 mL of 10% ethanol solution. Sugars were extracted by shaking at room temperature (25 °C) for 1 h, followed by centrifugation at 21,000 × g for 10 min with the supernatants stored at 4 °C and the pellets discarded. The supernatants containing free sugars were filtered through a 0.22 μm membrane and quantified by HPLC in a REZEX-ROA Organic acid H+ (8%) Ion exclusion column with a refractive index (RI) detector. Total starch content was determined according to the AOAC method 996.11^43^ using an amyloglucosidase/${\rm{\alpha }}$-amylase assay kit (Megazyme, Wicklow, Ireland). The supernatants containing hydrolysed starch were filtered through a 0.22 μm membrane and quantified by HPLC in a REZEX-ROA Organic acid H+ (8%) Ion exclusion column with a refractive index (RI) detector. Measurements were done once per experimental replicate. All values are reported as per cent dry matter basis (% DM).

### Phenolic compounds analysis

Phenolic compounds were extracted from the GJ and GS via solid-phase extraction (SPE). 50 mg of GS were extracted with 10 mL of 70% methanol (MeOH) aqueous solution and stored at −20 °C. 5 mL of GJ were centrifuged at 21,000 × *g* for 10 min and the supernatant stored at −20 °C. The stored fractions were passed through a Sep-Pak C18 Cartridge (Waters Ltd, Wilmslow, UK), conditioned with 4 mL of 100% MeOH, to bind the phenolic compounds, and then washed with 4 mL of 100% MeOH. The extracts were evaporated in an RC 10–22 centrifugal evaporator (Jouan, Saint Herblain, France) and the pellet resuspended in 200 μL of 70% MeOH, centrifuged at 21,000 × *g* for 10 min and the supernatants used for LC-MS analysis.

The SPE extracts containing phenolic compounds were quantified by LC-MS with a Nova-Pak C18 Column. Mobile phases comprised 0.1% formic acid aqueous solution (solvent A) and 0.1% formic acid MeOH solution (solvent B). Elution was performed from 0 to 50 min with 95–20% solvent A. Phenolic compounds were identified by comparison of each mass spectrum with spectra from authentic compounds analysed at Aberystwyth University, Aberystwyth. The approximate quantification was calculated from MS peak areas by multiplying the areas by the response factor of a Kaempferol calibration curve. The approximate concentration of all compounds was added up to a final total phenolic compound content (TPC).

### Amino acid profile analysis

Total AAs were prepared by mixing 200 mg of milled, dry SSF samples with 5 mL of 6 M HCl (0.1% phenol) in 15 mL glass tubes, degassed and sealed under an N_2_ stream, and incubated for 24 h at 110 °C and allowed to cool to room temperature. The hydrolysates were adjusted with 6 M NaOH to pH 6–8, made up to a final volume of 50 mL with water, and then centrifuged at 21,000 × *g* for 10 min, with the supernatant filtered through a 0.22 μm membrane and added in a 1:1 ratio to a 250 pmol/${\rm{\mu }}$L solution of DL-norvaline as an internal standard.

Pre-column derivatisation of amino acids with 6-aminoquinolyl-N-hydroxysuccinimidyl carbamate (AQC) was performed in accordance with Reverter, et al. ^45^. Briefly, 70 ${\rm{\mu }}$L of borate buffer was added to 10 ${\rm{\mu }}$L of AA hydrolysate, or amino acid standards, and 20 ${\rm{\mu }}$L of AQC solution (4 mg AQC in 1 mL anhydrous acetonitrile). After 1 min incubation at room temperature, the contents were transferred to an autosampler vial and capped with a silicone-lined septum. Derivatised samples were quantified by HPLC using an AccQ-Tag Amino acids C18 column with variable wavelength (VWD) (260 nm detection) and fluorescence (FLD) (266 nm emission and 473 nm detection) detectors. Mobile phases were composed of water (solvent A), acetonitrile (solvent B) and 50 mM ammonium formate (adjusted to pH 2.9 ± 0.05 with formic acid) (solvent C). Elution was carried out as follows: 0–0.5 min 0–1% solvent B and 100–99% solvent C, 0.5–15 min 1-4% solvent B and 99–96% solvent C, 15–23 min 4–5% solvent B and 96–95% solvent C, 23–24 min 5-9% solvent B and 95–91% solvent C, 24–28 min 9-14% solvent B and 91-86% solvent C, 28–45 min 14–17% solvent B and 86–83% solvent C, 45–55 min 17–60% solvent B and 83–0% solvent C. The flow rate was 1 mL/min, and the injection volume was 10 ${\rm{\mu }}$L. The AA in the samples were analysed according to retention time and peak area.

### Statistical analysis

The analytical data was analysed by two-tailed analysis of variance (ANOVA) followed by Tukey’s HSD post-hoc test using IBM SPSS v 28 statistical software (IBM UK Ltd, Hampshire, UK). The effect of the experimental treatments was analysed with a one-way ANOVA by comparing the results from each measurement at each time point (0, 24, 48 and 72 h) in two groups, BC/W and BC/GJ, and the different levels of BC/GJ/GS. The effect of time in the fermentation and the interaction between the experimental treatment and fermentation time was analysed by a two-way ANOVA in two separate groups, BC/W and BC/GJ (supplementary Table S2), and the different levels of BC/GJ/GS (Supplementary Table S3). Lastly, all experimental treatments at 72 h of SSF and those of unfermented BC were compared to identify the significant, most impactful changes with a one-way ANOVA followed by Tukey’s HSD post-hoc test (Supplementary Table S4). The results were expressed as mean value $\pm$ standard error. A statistical significance level (${\rm{\alpha }}$ 0.05) was used to analyse the results.

## Supplementary information

Supplementary Information

## Data Availability

The data supporting the findings reported herein are available on request from the corresponding author.
