# Supplementary material for: Improved nutritional value of surplus bread and perennial ryegrass via solid-state fermentation with Rhizopus oligosporus
Source: NPJ Sci Food. 2024 Nov 16;8:95. doi: 10.1038/s41538-024-00338-y (PMC11569167; doi:10.1038/s41538-024-00338-y)
Supplement: Supplementary file 1 — Supplementary Information [file 41538_2024_338_MOESM1_ESM.pdf]

Improved Nutritional Value of Surplus Bread and Perennial Ryegrass Via Solid-State Fermentation with *Rhizopus oligosporus*

Juan Felipe Sandoval, Joe Gallagher, Julia Rodriguez-Garcia, Kerry Whiteside, David N. Bryant

Supplementary materials

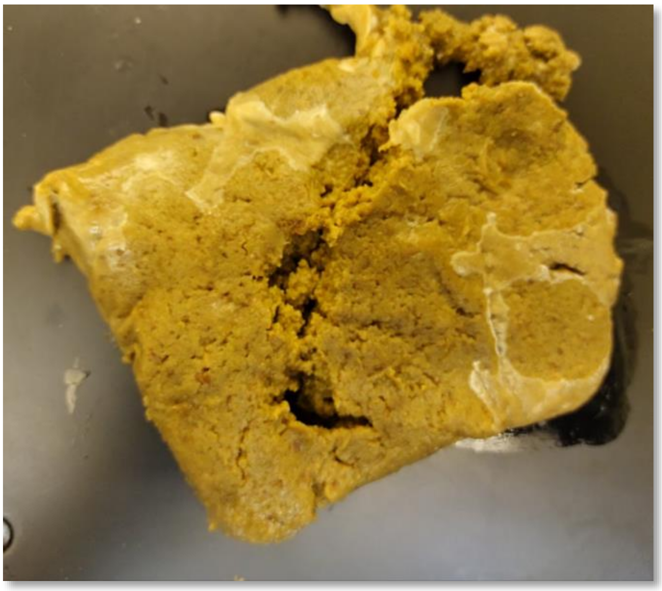

Figure S1. Photographic record SSF of bread crusts (BC)/water (W) with BC milled to a fine powder at 32 °C, moisture content of 56% and pH of 3.5 after 72 h. The fungi are not able to grow homogenously throughout the substrate

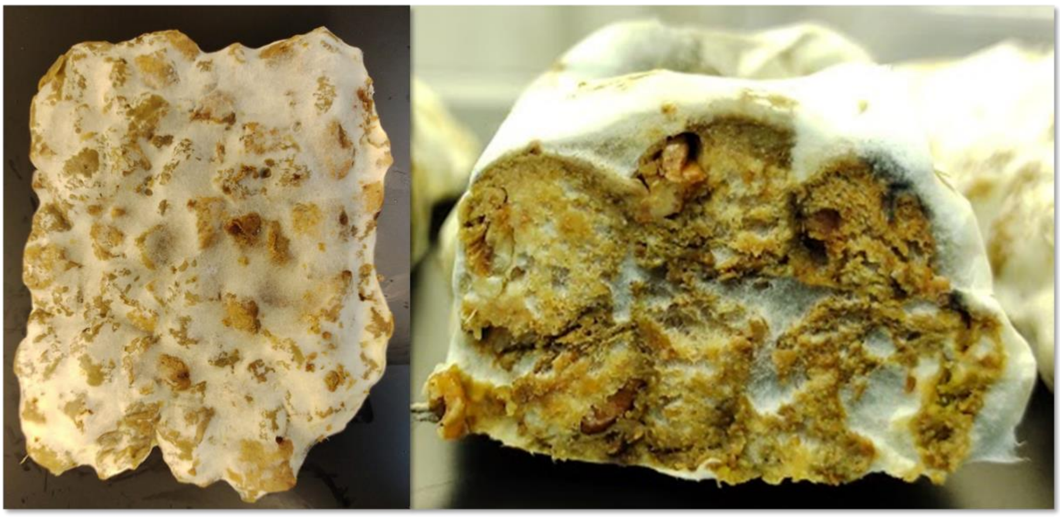

Figure S2. Photographic record SSF of bread crusts (BC)/water (W) with BC cut to 1x1x1 cm squares at 32 °C, moisture content of 56% and pH of 3.5 after 72 h. The fungi can grow homogeneously throughout the substrate

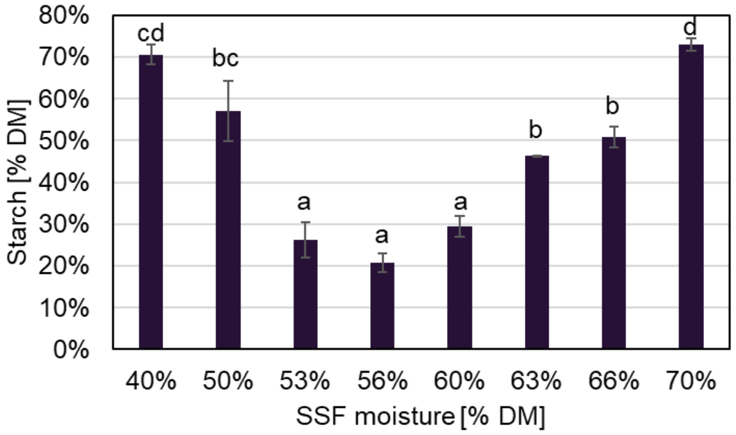

Figure S3. Final starch content of SSF of bread crusts (BC)/Grass Juice (GJ) with BC cut to 1x1x1 cm squares at 32 °C and pH of 3.5, at different starting SSF moisture levels, after 72 h. The error bars represent a standard error. Data with different letters are significantly different ($p < 0.05$), following Tukey´s HSD post-hoc test. The minimum value of starch is achieved similarly between 53 and 60% moisture content. 56% was chosen for all experiments for standardisation purposes.

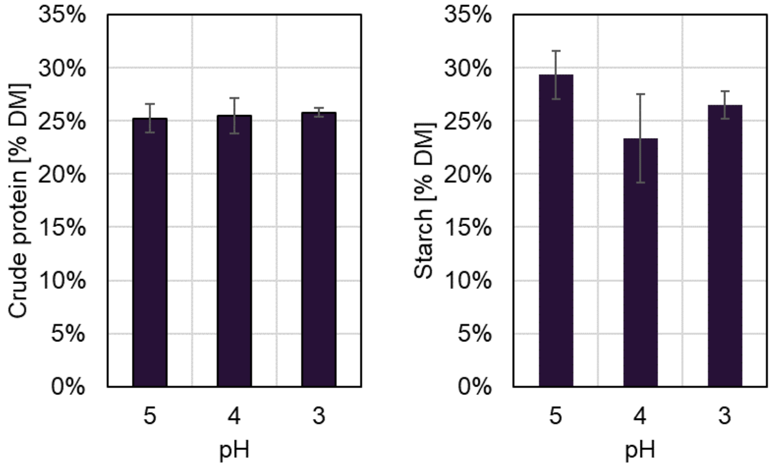

Figure S4. Final crude protein and starch content of SSF of bread crusts (BC)/Grass Juice (GJ) with BC cut to 1x1x1 cm squares at 32 °C and moisture content of 56%, at different starting pH, after 72 h. The error bars represent a standard error. No statistical difference was found between all starting pH points. 3.5 was chosen for all experiments for standardisation purposes and is low enough to avoid the growth of undesirable microorganisms.

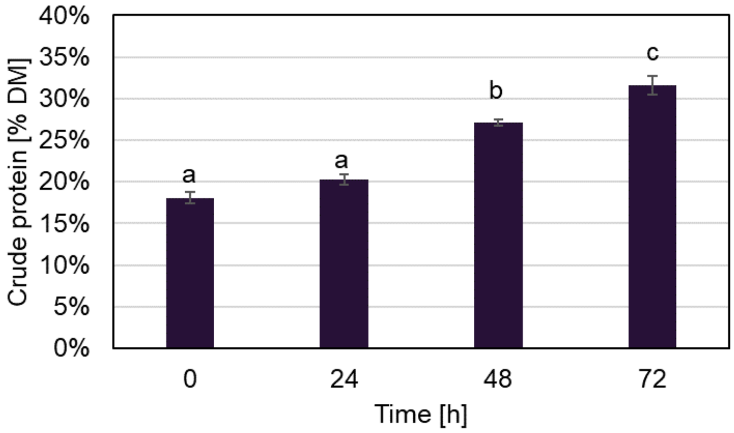

Figure S5. Crude protein content of bread crusts (BC)/water (W) and BC/green juice (GJ) experiments at 0, 24, 48 and 72 hours of SSF. The error bars represent a standard error.

Table S1. Total phenolic content (TPC) in grass juice (GJ) and grass solids (GS), and identified phenolic compounds by LC-MS. Results are expressed as the mean value ± a standard error.

Substrate	TPC [mg/g DM]	Identified phenolic compounds
Grass juice (GJ)	0.57 ± 0.01	Kaempferol, ferulic acid, apigenin, iso-rhamnetin, naringenin
Grass solids (GS)	5.21 ± 0.06	Kaempferol, p-coumaric acid, iso-rhamnetin

Table S2. Results of the statistical comparison of the effect of the experimental treatment (bread crusts (BC)/water (W) and BC/green juice (GJ)) and the fermentation time over multiple variables. Data shown are the p-values of a two-way ANOVA.

Result	Experimental treatment	Time	Experimental treatment * Time
SSF Mass loss [%]	0.000	0.000	0.158
**Chemical composition**			
Crude protein [%DM]	0.657	0.000	0.383
Crude fibre [%DM]	0.001	0.000	0.005
Free sugars [%DM]	0.597	0.000	0.004
Starch [%DM]	0.004	0.000	0.001
Ash [%DM]	0.000	0.000	0.002
Ratio of crude protein to starch	0.389	0.000	0.714
**EAA [%DM]**			
Histidine	0.908	0.000	0.002
Isoleucine	0.030	0.000	0.440
Leucine	0.945	0.000	0.549
Lysine	0.064	0.006	0.795
Methionine	0.001	0.138	0.246
Phenylalanine	0.000	0.000	0.030
Threonine	0.037	0.000	0.038
Valine	0.000	0.000	0.437

**NEAA [%DM]**			
Alanine	0.045	0.000	0.422
Aspartic acid	0.552	0.000	0.133
Cysteine	0.003	0.000	0.367
Glutamic acid	0.048	0.006	0.015
Glycine	0.000	0.000	0.000
Proline	0.000	0.384	0.029
Serine	0.013	0.000	0.040
Tyrosine	0.000	0.005	0.932
Arginine	0.039	0.000	0.039
**Sum AA [%DM]**	0.090	0.000	0.398
**Sum EAA [%DM]**	0.151	0.000	0.611
**Sum NEAA [%DM]**	0.065	0.000	0.208
**% EAA/AA**	0.485	0.000	0.195
**% NEAA/AA**	0.485	0.000	0.195

AA: Amino acids EAA: Essential amino acids, NEAA: Non-essential amino acids

Table S3. Results of the statistical comparison of the effect of the experimental treatment (bread crusts (BC)/ green juice (GJ)/dry green solids (GS) to crude protein 19%, 20%, 23%, 27% and 29% DM) and the fermentation time over multiple variables. Data shown are the p-values of a two-way ANOVA.

**Result**	**Experimental treatment**	**Time**	**Experimental treatment * Time**
SSF Mass loss [%]	0.000	0.000	0.189
**Chemical composition**			
Crude protein [%DM]	0.000	0.000	0.000
Crude fibre [%DM]	0.198	0.001	0.588
Free sugars [%DM]	0.000	0.000	0.015
Starch [%DM]	0.000	0.000	0.000
Ash [%DM]	0.003	0.000	0.496
Ratio of crude protein to starch	0.000	0.000	0.022
**EAA [%DM]**			
Histidine	0.000	0.004	0.001
Isoleucine	0.000	0.000	0.391
Leucine	0.000	0.001	0.391
Lysine	0.000	0.000	0.002
Methionine	0.000	0.001	0.721
Phenylalanine	0.000	0.002	0.001
Threonine	0.000	0.000	0.002
Valine	0.000	0.024	0.506
**NEAA [%DM]**			
Alanine	0.000	0.000	0.047
Aspartic acid	0.000	0.000	0.003
Cysteine	0.122	0.016	0.164
Glutamic acid	0.033	0.006	0.018
Glycine	0.152	0.075	0.319
Proline	0.015	0.851	0.267
Serine	0.000	0.000	0.003

Tyrosine	0.001	0.012	0.902
Arginine	0.000	0.000	0.000
**Sum AA [%DM]**	0.000	0.000	0.468
**Sum EAA [%DM]**	0.000	0.000	0.075
**Sum NEAA [%DM]**	0.000	0.001	0.503
**% EAA/AA**	0.000	0.010	0.036
**% NEAA/AA**	0.000	0.010	0.036

AA: Amino acids EAA: Essential amino acids, NEAA: Non-essential amino acids

Table S4. Results of the statistical comparison of the effect of the experimental treatment (bread crusts (BC)/water (W), BC/green juice (GJ) and BC/GJ/dry green solids (GS) to crude protein 19%, 20%, 23%, 27% and 29% DM) at 72 h of solid-state fermentation (SSF) versus unfermented BC over multiple variables. Data shown are the p-values of a one-way ANOVA and the grouping letters of a Tukey's HSD post-hoc analysis.

**Result**	**ANOVA p-value**	Tukey's HSD groups							
		**Unfermented BC**	**BC/W**	**BC/GJ**	**BC + GJ + GS to % DM CP**				
					**19 %**	**20 %**	**23 %**	**27 %**	**29 %**
**Chemical composition**									
Crude protein [%DM]	0.000	a	b	b	b	b	b	b	b
Crude fibre [%DM]	0.051	a	a	a	a	a	a	a	a
Free sugars [%DM]	0.000	c	c	b	bc	ab	ab	ab	a
Starch [%DM]	0.000	b	a	a	a	a	a	a	a
Ash [%DM]	0.000	a	bc	ab	bc	c	c	c	c
**Sum AA [%DM]**	0.000	a	bc	bcd	b	bc	d	cd	e
**Sum EAA [%DM]**	0.000	a	b	b	b	b	c	c	d
**Sum NEAA [%DM]**	0.000	a	bc	bc	ab	bc	c	bc	d
**% EAA/AA**	0.000	a	bc	bc	ab	bc	c	bc	d
**% NEAA/AA**	0.000	a	bc	bc	ab	bc	cd	d	cd

AA: Amino acids EAA: Essential amino acids, NEAA: Non-essential amino acids
